# The Rhizome Mixture of* Anemarrhena asphodeloides* and* Coptis chinensis* Attenuates Mesalazine-Resistant Colitis in Mice

**DOI:** 10.1155/2016/5895184

**Published:** 2016-09-27

**Authors:** Su-Min Lim, Hyun Sik Choi, Jin-Ju Jeong, Seung-Won Han, Dong-Hyun Kim

**Affiliations:** ^1^Department of Life and Nanopharmaceutical Sciences and Department of Pharmacy, 26 Kyungheedae-ro, Dongdaemun-gu, Seoul 02447, Republic of Korea; ^2^DongWha Pharm Research Institute, 35-71 Topsil-ro, Giheung-gu, Yongin-Shi, Gyeonggi 446-902, Republic of Korea

## Abstract

We investigated the effect of DWac on the gut microbiota composition in mice with 2,3,6-trinitrobenzenesulfonic acid- (TNBS-) induced colitis. Treatment with DWac restored TNBS-disturbed gut microbiota composition and attenuated TNBS-induced colitis. Moreover, we examined the effect of DWac in mice with mesalazine-resistant colitis (MRC). Intrarectal injection of TNBS in MRC mice caused severe colitis, as well as colon shortening, edema, and increased myeloperoxidase activity. Treatment with mesalazine (30 mg/kg) did not attenuate TNBS-induced colitis in MRC mice, whereas treatment with DWac (30 mg/kg) significantly attenuated TNBS-induced colitis. Moreover, treatment with the mixture of mesalazine (15 mg/kg) and DWac (15 mg/kg) additively attenuated colitis in MRC mice. Treatment with DWac and its mixture with mesalazine inhibited TNBS-induced activation of NF-*κ*B and expression of M1 macrophage markers but increased TNBS-suppressed expression of M2 macrophage markers. Furthermore, these inhibited TNBS-induced T-bet, ROR*γ*t, TNF-*α*, and IL-17 expression but increased TNBS-suppressed Foxp3 and IL-10 expression. However, Th2 cell differentiation and GATA3 and IL-5 expression were not affected. These findings suggest that DWac can ameliorate MRC by increasing the polarization of M2 macrophage and correcting the disturbance of gut microbiota and Th1/Th17/Treg, as well as additively attenuating MRC along with mesalazine.

## 1. Introduction 

Inflammatory bowel disease (IBD), which includes ulcerative colitis and Crohn's disease, is a chronic, remitting-relapsing, inflammatory disease [1, 2]. Approximately, 15% of patients with IBD develop severe disease [3]. As part of IBD's pathogenesis, intestinal lymphoid cells, dendritic cells, macrophages, and T cells in the inflamed gastrointestinal tract increase the expression of inflammatory mediators, such as tumor necrosis factor- (TNF-) *α* and interleukin- (IL-) 17. These inflammatory mediators play a pivotal role in the initiation, progression, and persistence of chronic inflammation [4–7]. IL-17 produced by T-helper cells is a cytokine that recruits neutrophils and monocytes to the site of inflammation [8] and acts synergistically with TNF-*α* [9, 10]. IL-10 stimulates Treg cell differentiation and antagonizes Th17 cells [11]. The number of Th17 cells, which produce IL-17 and IL-22 and activate M1 macrophages, which secrete TNF-*α* and IL-6, is greater in patients with IBD than in healthy controls [12]. Therefore, therapeutic approaches to suppress IL-17 and TNF-*α* expression and to induce IL-10 expression are helpful for managing chronic colitis [13]. Based on these findings, to induce remission from ulcerative colitis, oral or topical 5-aminosalicylates, which inhibit macrophage activation involved in innate immunity and polarize M1 macrophages to M2 macrophages, are frequently used with or without corticosteroids. To maintain remission, 5-aminosalicylates such as mesalazine and immunomodulators such as azathioprine and 6-mercaptopurine are used [3]. If 5-aminosalicylates do not induce remission, TNF-*α* antibodies such as infliximab are used [14] however, these drugs have the potential to cause severe side effects and are very expensive.

The rhizome mixture of* Anemarrhena asphodeloides* and* Coptis chinensis* (DWac) exhibits an anti-inflammatory effect in mice with 2,4,6-trinitrobenzenesulfonic acid- (TNBS-), dextran sulfate sodium- (DSS-), or oxazolone-induced colitis by inhibiting NF-*κ*B and MAPK signaling pathways and correcting disturbed Th17/Treg cells [15, 16]. However, the effect of DWac against gut microbiota disturbance and mesalazine-resistant colitis has not been studied.

In order to investigate whether DWac can restore the disturbed gut microbiota in colitis and induce remission in mesalazine-resistant colitis (MRC), we investigated the effect of DWac on the gut microbiota composition in mice with 2,4,6-trinitrobenzenesulfonic acid- (TNBS-) induced colitis by pyrosequencing and the anti-inflammatory effect of DWac in mice with mesalazine-resistant colitis.

## 2. Materials and Methods

### 2.1. Materials

TNBS was purchased from Sigma (St. Louis, MO, USA). Protease inhibitor cocktail was purchased from Roche Applied Science (Roche, Mannheim, Germany). Antibodies were purchased from Cell Signaling (Danvers, MA, USA). Enzyme-linked immunosorbent assay (ELISA) kits were purchased from R&D Systems (Minneapolis, MN, USA). The enhanced chemiluminescence (ECL) immunoblot system was purchased from Pierce Co. (Rockford, IL, USA).

DWac containing 1.07% mangiferin and 4.38% berberine was donated from DongWha Pharm Co. (Seoul, Korea).

### 2.2. Animals

Male C57BL/6 (20–23 g, 6 weeks old) were supplied from RaonBio (Seoul, Korea) and maintained at an ambient temperature of 22°C ± 1°C and humidity of 50% ± 10% (lights on 07:00–19:00) for 7 days before using the experiment. Mice were provided with water and food ad libitum. All animal experiments, approved by the Committee for the Care and Use of Laboratory Animals in Kyung Hee University (KHP-2014-04-02), were performed according to the Kyung Hee University guideline for Laboratory Animals Care and Use.

### 2.3. Preparation of Mice with TNBS-Induced Colitis and Induction of MRC

First, to obtain a model for TNBS-induced colitis, mice were randomly divided into three (for the analysis of gut microbiota composition) or eight groups (for the analysis of dose-dependency). To understand the effect of DWac on the gut microbiota composition, colitis was induced by intrarectal injection of 0.1 mL of 2.5% (w/v) TNBS solution according to the method of Lim et al. [16]. Each group consisted of five mice. The normal group was treated with saline instead of TNBS solution. DWac (20 mg/kg) dissolved in saline was orally administered once a day for three days starting 24 h after TNBS injection.

Next, for the preparation of mice with MRC, mice were randomly divided into six groups: one normal, one TNBS alone-treated colitis group, and four MRC groups treated with vehicle alone (TM), DWac (TM-D, 30 mg/kg), mesalazine (TM-M, 30 mg/kg), or their mixture (TM-MD, the mixture of 15 mg/kg DWac and 15 mg/kg mesalazine). Each group consisted of six mice. MRC was induced by the intermittent intrarectal injection of TNBS and mesalazine as follows. First 0.1 mL of 1.25% (w/v) TNBS solution dissolved in 50% ethanol was intrarectally administered one, after which mesalazine was orally administered once a day for three days. This was repeated for three rounds. After fourth treatment with TNBS, the test agents (TM, vehicle alone; TM+M, 30 mg/kg; TM-D, 30 mg/kg DWac; or TM-MD, the mixture of 15 mg/kg DWac and 15 mg/kg mesalazine) were orally administered once a day for three days. The TM group was treated with TNBS alone in mice repeated with TNBS and mesalazine for three rounds. The mice were killed 18 h after the final administration of the test agents. The colon was quickly removed, opened longitudinally, and gently washed with phosphate-buffered saline on ice. Macroscopic scores for colitis severity were assessed according to the method of Lim et al. [16]. The colons were then excised, treated with ice-cold perfusion solution (0.15 M KCl, 2 mM EDTA, pH 7.4), homogenized in 50 mM Tris-HCl buffer (pH 7.4), and centrifuged at 10,000 ×g and 4°C for 30 min [16]. The resulting supernatants were stored at −80°C until used in ELISA, immunoblotting, and myeloperoxidase activity assays.

### 2.4. Myeloperoxidase Activity Assay

The supernatant of colon homogenates (50 *μ*L) was added to a reaction mixture containing 0.1 mM H_2_O_2_ and 1.6 mM tetramethyl benzidine, incubated at 37°C for 2 min, and monitored the absorbance at 650 nm time-over [16].

### 2.5. ELISA and Immunoblotting

For the determination of cytokines, colon supernatants were transferred to 96-well ELISA plates. The cytokine levels were assayed using commercial ELISA kits (Pierce Biotechnology, Inc., Rockford, IL, USA) [16].

For immunoblotting, the supernatants were subjected to sodium dodecyl sulfate-polyacrylamide gel electrophoresis, then transferred to nitrocellulose membrane, and immunodetected using an enhanced chemiluminescence detection kit according to the method of Lim et al. [16].

### 2.6. Quantitative Real Time-Polymerase Chain Reaction (qPCR)

For the assay of arginase 1, arginase 2, TNF-*α*, IL-1*β*, IL-10, CD206, T-bet, GATA3, ROR*γ*t, Foxp3, INF-*γ*, IL-5, IL-17, and GAPDH expressed in the colon, qPCR was performed according to the method of Kim et al. [17] and Lim et al. [18], utilizing Takara thermal cycler, which used SYBER premix agents, as per the instructions from Takara biology incorporation: activation of DNA polymerase at 95°C for 5 min and 32 cycles of amplification at 95°C for 10 s and at 60°C for 30 s. The amounts of expressed genes were calculated, with respect to GAPDH, using the Microsoft Excel data spreadsheet.

### 2.7. Gene Pyrosequencing

Genomic DNA was extracted from the fresh feces of mice using a commercial DNA isolation kit (QIAamp DNA stool mini kit, Hilden, Germany) [17] and amplified using barcoded primers (the V1 to V3 region of the bacterial 16S rRNA gene). Pyrosequencing was performed by a 454 GS FLX Titanium Sequencing System (Roche, Branford, CT) and identified using the EzTaxon-e database (http://eztaxon-e.ezbiocloud.net/) [17]. The number of sequences analyzed, observed diversity richness (operational taxonomic units, OTUs), estimated OTU richness (ACE and Chao1), and coverage are shown in Table 1.

### 2.8. Statistical Analysis

Data were expressed as the mean ± standard deviation and analyzed by one-way analysis of variance (ANOVA) followed by the Student-Newman-Keuls test for multiple comparisons. *p* values of 0.05 or less were considered statistically significant.

## 3. Results

**Figure 1:**
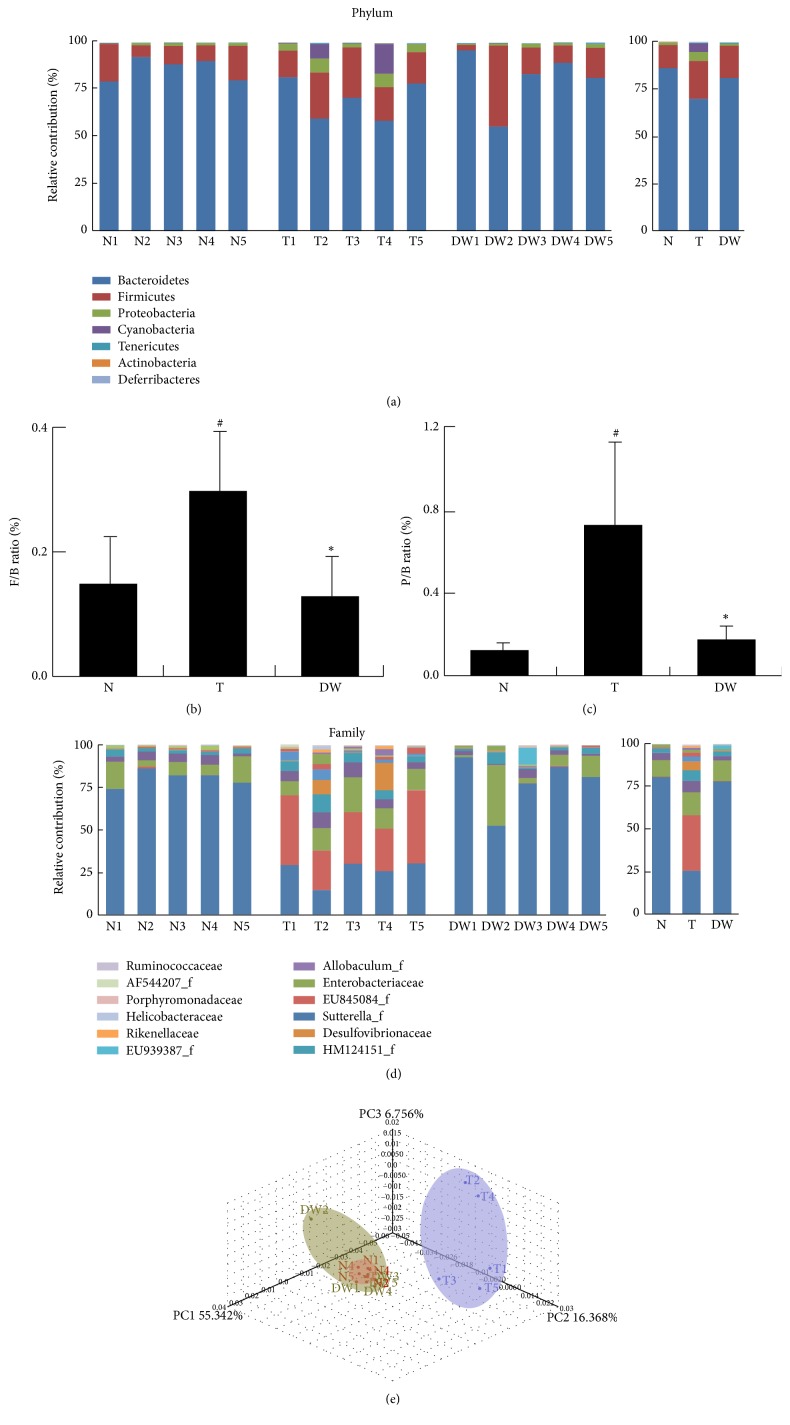
Effect of DWac on the gut microbiota composition in mice with TNBS-induced colitis. (a) Effect on phylum level. (b) The ratio of* Firmicutes* to* Bacteroidetes* (*n* = 5). (c) The ratio of* Proteobacteria *to* Bacteroidetes* (*N* = 5). (d) Effect on family level. Genomic DNA was extracted from the fecal samples taken from mice treated with vehicle alone (N1~N5), TNBS alone (T1~T5), or DWac (20 mg/kg) in the presence of TNBS (DW1~DW5) and analyzed by pyrosequencing of the bacterial 16S rRNA fragments (*n* = 5). (e) Principal coordinate analysis (PCoA) plot. The plot shows the clustering pattern among mice treated with vehicle alone (N1~N5), TNBS alone (T1~T5), or DWac in the presence of TNBS (DW1~DW5) based on weighted pairwise Fast UniFrac analysis. ^#^ *p* < 0.05 versus N (normal) group. ^*∗*^ *p* < 0.05 versus T (TNBS) group.

In a previous study, DWac was shown to have an anticolitic effect in mice with TNBS-induced colitis by inhibiting macrophage activation and correcting disturbed Th17/Treg differentiation [16, 18]. However, the effect of DWac on the gut microbiota composition has not been studied. Therefore, we investigated its effect on the gut microbiota composition in mice with TNBS-induced colitis using pyrosequencing (Figure 1). Comparing the results of taxonomy-based analysis between mice with and without TNBS-induced colitis, treatment with TNBS caused a significant modulation of the populations. Treatment with TNBS induced an increase in Firmicutes and Proteobacteria phyla and a decrease in Bacteroidetes; as a result, TNBS increased the ratio of Firmicutes or proteobacteria to Bacteroidetes. Oral administration of DWac restored TNBS-induced ratio of Firmicutes or Proteobacteria to Bacteroidetes. At the family level, treatment with DWac restored TNBS-induced number of Prevotellaceae, Ruminococcaceae, and AF544207_f and TNBS-suppressed number of EF602759_f* Prevotellaceae* but decreased the numbers of* Turicibacter_f*. Next, we processed all these sequences to match the length and position of the 16S rRNA gene sequences of gut microbiota, computed all pair-wise distances among mice treated with vehicle, TNBS alone, and DWac with TNBS, and performed principal coordinate analysis (PCoA). The gut microbial community of mice treated with TNBS alone was significantly different from that of mice treated without TNBS. Treatment with DWac restored TNBS-disturbed gut microbiota composition and attenuated TNBS-induced colitis. There were no significant differences in bacterial richness and diversity between the fecal samples of the mice (Table 1).

**Figure 2:**
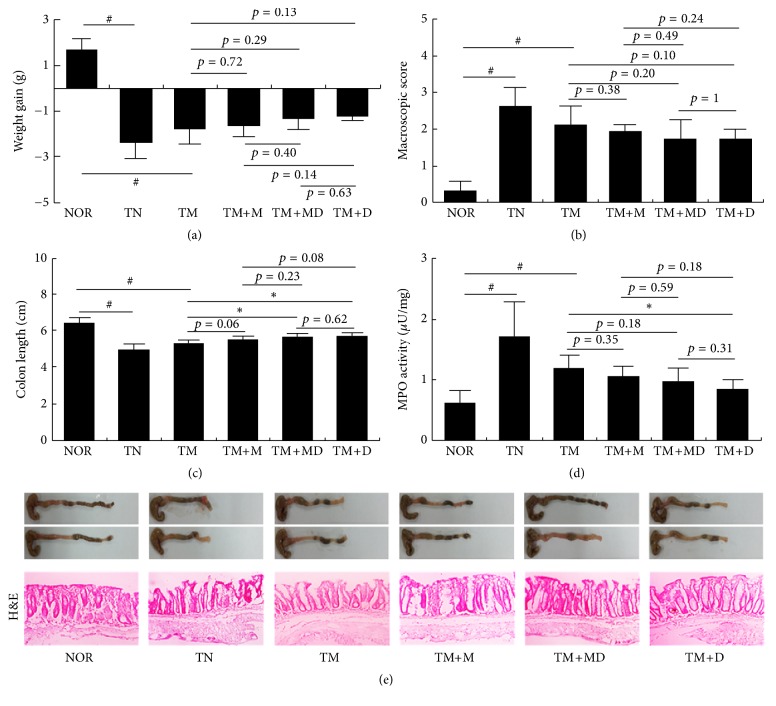
Effect of DWac and mesalazine on body weight (a), macroscopic disease (b), colon length (c), colonic myeloperoxidase (MPO) activity (d), and histological exam (e) in mesalazine-resistant mice. Mesalazine-resistant colitis (MRC) was induced by the repeated intrarectal injection of TNBS and oral administration of mesalazine. MRC mice, except normal control group, were orally treated with saline (TM) or test agents (TM+M, 30 mg/kg mesalazine; TM+DM, the mixture of 15 mg/kg DWac and 15 mg/kg mesalazine; TM+D, 30 mg/kg DWac) for 3 days after TNBS treatment. TN was treated 4 times with TNBS alone and vehicle. The mice were killed 18 h after the final administration of test agents. All data are mean ± SD (*n* = 6). ^#^ *p* < 0.05 versus control group. ^*∗*^ *p* < 0.05 versus TM group.

**Figure 3:**
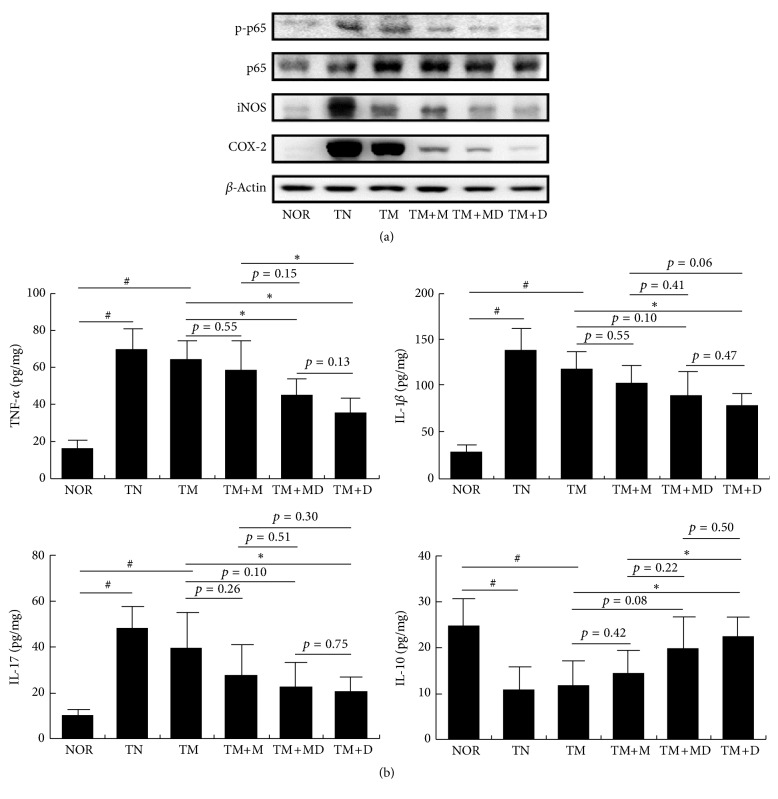
Effects of DWac and mesalazine on the expression of iNOS and COX-2, activation of NF-*κ*B (a), and expression of inflammatory cytokines (b) in mice with MRC. Mesalazine-resistant colitis (MRC) was induced by the repeated intrarectal injection of TNBS and oral administration of mesalazine. MRC mice, except normal control group, were orally treated with saline (TM) or test agents (TM+M, 30 mg/kg mesalazine; TM+DM, the mixture of 15 mg/kg DWac and 15 mg/kg mesalazine; TM+D, 30 mg/kg DWac) for 3 days after TNBS treatment. TN was treated 4 times with TNBS alone and vehicle. The mice were killed 18 h after the final administration of test agents. iNOS, COX-2, and NF-*κ*B were determined by immunoblotting. Cytokines were determined by ELISA. All data are mean ± SD (*n* = 6). ^#^ *p* < 0.05 versus control group. ^*∗*^ *p* < 0.05 versus TM group.

Next, we induced MRC in mice by repeatedly treating mesalazine and inducing colitis with TNBS. The repeated treatment caused severe colitis, concurrent with colon shortening, edema, and increase of myeloperoxidase activity (Figure 2). The inflammatory parameters, such as body weight, colon length, and myeloperoxidase activity, in mice with colitis induced by the repetitive intrarectal injection of TNBS and repetitive treatment with mesalazine were not significantly different from those with colitis induced by a single treatment with TNBS. However, treatment with mesalazine was not effective. Thus, treatment with mesalazine did not suppress NF-*κ*B activation or iNOS and COX-2 expression. Therefore, we defined it to be MRC (Figure 3). However, treatment with DWac suppressed colon shortening and myeloperoxidase activity and also inhibited iNOS and COX-2 expression, as well as NF-*κ*B activation in mice with MRC. DWac treatment also suppressed TNF-*α*, IL-1*β*, and IL-17 expression but increased IL-10 expression. Furthermore, we found that treatment with the mixture (1 : 1, w/w) of DWac and mesalazine (30 mg/kg) also ameliorated colon length, macroscopic score, and myeloperoxidase activity in mice with MRC. Treatment with the mixture also suppressed iNOS and COX-2 expression and NF-*κ*B activation in mice with MRC.

**Figure 4:**
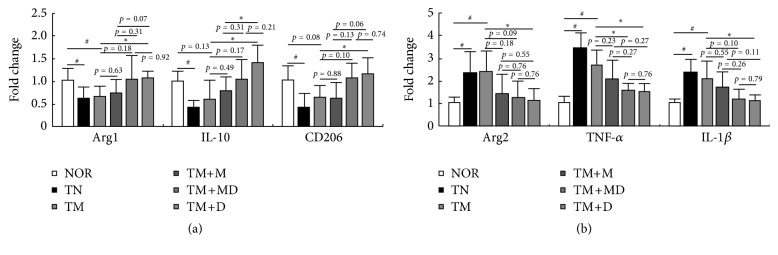
Effects of DWac and mesalazine on the expression of M1 and M2 macrophage markers in mice with MRC. Mesalazine-resistant colitis (MRC) was induced by the repeated intrarectal injection of TNBS and oral administration of mesalazine. MRC mice, except normal control group, were orally treated with saline (TM) or test agents (TM+M, 30 mg/kg mesalazine; TM+DM, the mixture of 15 mg/kg DWac and 15 mg/kg mesalazine; TM+D, 30 mg/kg DWac) for 3 days after TNBS treatment. TN was treated 4 times with TNBS alone and vehicle. The mice were killed 18 h after the final administration of test agents. M1 and M2 macrophage markers were assayed by qPCR. All data are mean ± SD (*n* = 6). ^#^ *p* < 0.05 versus control group. ^*∗*^ *p* < 0.05 versus TM group.

To investigate the anti-inflammatory effect of DWac and its mixture with mesalazine on the macrophage polarization related to the innate immune response, we examined the effect of DWac on macrophage polarization in mice with MRC (Figure 4). Treatment with TNBS in mice with MRC significantly induced the expression of M1 macrophage markers such as arginase 2, IL-1*β*, and TNF-*α* but suppressed the expression of M2 macrophage markers, such as arginase 1, IL-10, and CD206. These cytokines such as TNF-*α* and IL-10 produce various immune cells, including macrophages and T cells [19]. Nevertheless, these cytokines secreted in these immune cells regulated macrophage polarization. Oral administration of DWac significantly inhibited TNBS-induced expression of arginase 2, IL-1*β*, and TNF-*α*, whereas treatment with the mixture of DWac and mesalazine inhibited only the expression of TNF-*α* alone. However, treatment with mesalazine did not significantly affect the expression of tested M1 macrophage markers. Furthermore, DWac and its mixture with mesalazine significantly increased IL-10 expression, whereas arginase 1 and CD206 were significantly increased by DWac alone.

**Figure 5:**
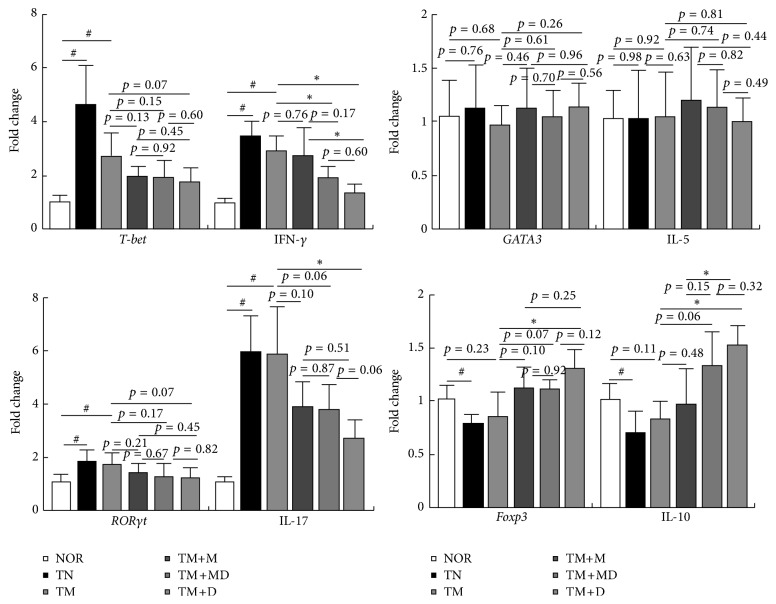
Effects of DWac and mesalazine on the expression of transcription factors and cytokines of Th1, Th2, Th17, and Treg cells in mice with MRC. Mesalazine-resistant colitis (MRC) was induced by the repeated intrarectal injection of TNBS and oral administration of mesalazine. MRC mice, except normal control group, were orally treated with saline (TM) or test agents (TM+M, 30 mg/kg mesalazine; TM+DM, the mixture of 15 mg/kg DWac and 15 mg/kg mesalazine; TM+D, 30 mg/kg DWac) for 3 days after TNBS treatment. TN was treated 4 times with TNBS alone and vehicle. The mice were killed 18 h after the final administration of test agents. Transcription factors and cytokines were assayed by qPCR. All data are mean ± SD (*n* = 6). ^#^ *p* < 0.05 versus control group. ^*∗*^ *p* < 0.05 versus TM group.

Next, we examined the effect of DWac and its mixture with mesalazine on the transcription factors and cytokines of T cells in mice with MRC (Figure 5). Treatment with TNBS/mesalazine in mice with MRC significantly induced the expression of T-bet, ROR*γ*t, IFN*γ*, and IL-17 and suppressed the expression of Foxp3 and IL-10. However, TNBS did not affect GATA3 and IL-5. Oral administration of DWac and its mixture with mesalazine significantly inhibited TM-induced expression of T-bet, ROR*γ*t, IFN*γ*, and IL-17, whereas these increased TM-suppressed expression of Foxp3 and IL-10. However, treatment with mesalazine alone did not significantly affect the expression of these transcription factors and cytokines. Furthermore, DWac, mesalazine, and their mixture did not affect the expression of GATA3 and IL-5.

## 4. Discussion

DWac have been shown to attenuate TNBS- and DSS-induced colitis, as well as collagen-induced arthritis in mice by inhibiting the NF-*κ*B signaling pathway and correcting Th17/Treg disturbance in vivo. DWac also inhibited the expression of TNF-*α* and IL-6 in LPS-stimulated macrophages by inhibiting IRAK1 phosphorylation and LPS binding to TLR4 in vitro [15, 16]. Furthermore, DWac inhibited the differentiation of splenic Th cells to Th17 cells by suppressing ROR*γ*t and IL-17 expression but increased differentiation to Treg cells by inducing Foxp3 and IL-10 expression. The anticolitic effect of DWac was comparable to that of mesalazine, which is a well-known commercial drug used for colitis [20]. Furthermore, in the present study, we found that TNBS treatment resulted in a significant increase in Proteobacteria and Firmicutes, as well as a reduction in Bacteroidetes on the gut microbiota composition in mice. DWac treatment restored TNBS-disturbed gut microbiota composition and attenuated TNBS-induced colitis. DWac suppressed TNBS-induced expression of M1 macrophage markers but increased M2 macrophage marker expression. These results suggest that DWac can ameliorate colitis by regulating gut microbiota composition, including macrophage activation in the innate immunity and Th cell differentiation in the adaptive immunity.

In the present study, we developed the inceptive MRC mouse model. This model significantly activated innate and adaptive immune responses, like a mouse model previously developed by TNBS (Table 2). Moreover Th17 transcription factor ROR*γ*t and its cytokine IL-17 were more potently expressed in MRC mouse model than in a mouse model with TNBS-induced colitis. First of all, MRC mouse model was mesalazine-resistant. In MRC mouse model, DWac attenuated TNBS-induced colitis by inhibiting the NF-*κ*B signaling pathway. When DWac was mixed with mesalazine, the mixture also exhibited an anticolitic effect in vivo but was more effective than mesalazine alone. These results suggest that DWac may synergistically add to the anticolitic effect of mesalazine. Sulfasalazine and mesalazine are frequently used as remissive drugs for ulcerative colitis and can prevent the relapse of ulcerative colitis due to resistance to aminosalicylates in 65–80% of patients [3, 14]. Developing therapeutic modalities that induce remission in the remaining patients is important in the treatment of IBD and the combination of 5-aminosalicylic acid derivatives and corticosteroids, such as prednisolone, immunomodulators, such as azathioprine and 6-mercaptopurine, or TNF-*α* antibody drugs, such as infliximab, is used. DWac and its mixture with mesalazine both attenuated MRC.

## 5. Conclusion

DWac restored TNBS-disturbed gut microbiota composition in mice and attenuated TNBS-induced colitis. Oral administration of DWac (30 mg/kg) significantly attenuated TNBS-induced colitis with MRC. Moreover, the mixture of mesalazine (15 mg/kg) and DWac (15 mg/kg) additively attenuated colitis in MRC mice. DWac and the mixture inhibited TNBS-induced activation of NF-*κ*B and expression of M1 macrophage markers but increased TNBS-suppressed expression of M2 macrophage markers. These also inhibited TM-induced T-bet, ROR*γ*t, IFN-*γ*, and IL-17 and increased TN-induced Foxp3 and IL-10. These findings suggest that DWac can ameliorate MRC by increasing M2 macrophage polarization and correcting the disturbance of gut microbiota and Th1/Th17/Treg; therefore, we suggest DWac may become a promising drug for patients with mesalazine-sensitive and mesalazine-resistant colitis.

## Figures and Tables

**Table 1:** Number of sequences analyzed, observed diversity richness, estimated operational taxonomic units (OTUs) richness for abundance-based coverage estimator (ACE) and Chao1, and coverage.

Sample	Valid reads	OTUs	Ace	Chao1	Goods lib. coverage
N1	3121	399	876.00	722.77	0.94
N2	3510	427	624.78	652.42	0.95
N3	3973	457	981.59	777.97	0.95
N4	2994	385	796.42	650.68	0.94
N5	4016	456	795.83	677.26	0.96

Mean ± SD	3523 ± 421	425 ± 29	814.92 ± 116.94	696.22 ± 48.45	0.95 ± 0.01

T1	11022	750	1151.43	1182.11	0.97
T2	8261	654	1132.29	989.56	0.97
T3	8800	811	1547.27	1251.51	0.96
T4	9026	773	1466.54	1207.28	0.96
T5	8659	744	1534.78	1242.03	0.96

Mean ± SD	9153 ± 967	746 ± 52	1366.46 ± 185.53	1174.50 ± 95.74	0.97 ± 0.01

DW1	3775	377	653.11	549.08	0.96
DW2	5075	717	1220.93	1059.50	0.95
DW3	4626	477	855.98	738.26	0.96
DW4	3702	441	648.51	658.50	0.95
DW5	4625	532	978.21	835.88	0.95

Mean ± SD	4361 ± 534	509 ± 116	871.35 ± 215.01	768.25 ± 173.42	0.95 ± 0.01

**Table 2:** Properties of mice with TNBS-induced colitis caused by treatment with a single or repetitive TNBS in the absence or presence of mesalazine.

Parameter	TC (TNBS)^a^	MTC (modified TNBS)^b^	MRC (modified TNBS plus mesalazine)^c^
Myeloperoxidase	++^d^	+	+
TNF-*α*	++	+	+
IL-1*β*	++	+	+
IFN*γ*	++	++	++
IL-5	−	0	0
IL-10	−	−	−
IL-17	+	++	++
T-bet	+	++	+
GATA3	−	0	0
ROR*γ*t	++	++	+
Foxp3	−	−	−
Mesalazine resistance	0	0	++

^a^Mice with TNBS-induced colitis (TC) were intrarectally treated with TNBS (0.2 mL of 5% (w/v) TNBS solution) at once.

^b^Mice with modified TNBS-induced colitis (MTC) were intrarectally treated with TNBS (0.1 mL of 2.5% (w/v) TNBS solution) four times.

^c^Mice with MRC were intrarectally treated with TNBS (0.1 mL of 2.5% (w/v) TNBS solution) four times and mesalazine three times.

^d^Expression levels of parameters are indicated as follows: 0, no induction or no suppression; +, moderate induction; ++, strong induction; −, moderate suppression.
